# An efficient magnetically modified microbial cell biocomposite for carbazole biodegradation

**DOI:** 10.1186/1556-276X-8-522

**Published:** 2013-12-11

**Authors:** Yufei Li, Xiaoyu Du, Chao Wu, Xueying Liu, Xia Wang, Ping Xu

**Affiliations:** 1 State Key Laboratory of Microbial Technology, Shandong University, Jinan 250100, People’s Republic of China; 2 Present address: State Key Laboratory of Microbial Metabolism, and School of Life Sciences & Biotechnology, Shanghai Jiao Tong University, Shanghai 200240, People’s Republic of China

**Keywords:** Carbazole, Immobilization, Nanoparticles, Biodegradation, Reusability

## Abstract

Magnetic modification of microbial cells enables to prepare smart biocomposites in bioremediation. In this study, we constructed an efficient biocomposite by assembling Fe_3_O_4_ nanoparticles onto the surface of *Sphingomonas* sp. XLDN2-5 cells. The average particle size of Fe_3_O_4_ nanoparticles was about 20 nm with 45.5 emu g^-1^ saturation magnetization. The morphology of *Sphingomonas* sp. XLDN2-5 cells before and after Fe_3_O_4_ nanoparticle loading was verified by scanning electron microscopy and transmission electronic microscopy. Compared with free cells, the microbial cell/Fe_3_O_4_ biocomposite had the same biodegradation activity but exhibited remarkable reusability. The degradation activity of the microbial cell/Fe_3_O_4_ biocomposite increased gradually during recycling processes. Additionally, the microbial cell/Fe_3_O_4_ biocomposite could be easily separated and recycled by an external magnetic field due to the super-paramagnetic properties of Fe_3_O_4_ nanoparticle coating. These results indicated that magnetically modified microbial cells provide a promising technique for improving biocatalysts used in the biodegradation of hazardous compounds.

## Background

As types of toxic and mutagenic common nitrogen compounds, carbazole and its derivatives readily undergo radical chemistry to generate the more poisonous hydroxynitrocarbazoles [1-4]. Soil, river sediments, and ground water polluted by carbazole have become a great threat to the environment. Therefore, it is necessary to establish effective methods to clear up carbazole and its derivatives.

Nanoscale iron particles represent a new generation of environmental remediation technologies that could provide cost-effective solutions to some of the most challenging environmental cleanup problems [5]. Due to biocompatibility, large surface areas, high surface reactivity, and super-paramagnetic properties, nanoscale iron particles provide enormous flexibility for environmental applications [6-8]. Research has shown that nanoscale iron particles are very effective for the transformation and detoxification of a wide variety of common environmental contaminants, such as hazardous organic compound [9-11] and heavy metal ions [8,12].

The use of immobilized microorganisms rather than free cells in biodegradation can be advantageous to enhance the stability of the biocatalyst and to facilitate its recovery and reuse. Entrapment method as a traditional method is widely used in the immobilization of microorganisms [13]. In our previous study, *Sphingomonas* sp. XLDN2-5 as a carbazole-degrading strain was entrapped in the mixture of Fe_3_O_4_ nanoparticles and gellan gum using modified traditional entrapment method [7]. However, the mass-transfer problems of limited diffusion and steric hindrance reduced microbial cell access to substrate [14]. Therefore, we constructed an efficient biocomposite by assembling Fe_3_O_4_ nanoparticles onto the surface of *Sphingomonas* sp. XLDN2-5 cells in this study. The resulting microbial cell/Fe_3_O_4_ biocomposite exhibited good biodegradation activity and reusability.

## Methods

Analytical grade carbazole was purchased from Sigma-Aldrich (St. Louis, MO, USA). All other chemicals were of analytical grade and commercially available.

*Sphingomonas* sp. XLDN2-5, which can use carbazole as the sole source of carbon, nitrogen, and energy, was cultivated in the mineral salts medium (MSM) as previously described [15]. Cells were harvested in the exponential phase (the optical density was about 0.68 to 0.70 at 620 nm) by centrifugation at 12,000 rpm for 10 min. The pellet was washed thrice with sodium chloride solution (0.9%, *w*/*v*) and then resuspended in sodium chloride solution (0.9%, *w*/*v*).

Fe_3_O_4_ nanoparticles were prepared as previously described [7]. Fe_3_O_4_ powder (1.0 g) was put into 100 ml distilled water to form the Fe_3_O_4_ particle suspension. After ultrasonic disruption (25 KHz, 10 min; BUG25-06, Branson, MO, USA) of the suspension, the Fe_3_O_4_ nanoparticles were well dispersed in distilled water to form a stable suspension.

Fe_3_O_4_ particle suspension (1%, *w*/*v*) and cell suspension were mixed with the ratio of cell wet weight to Fe_3_O_4_ of 1 (*w*/*w*). Microbial cells and Fe_3_O_4_ nanoparticles were fully mixed by vortexing, then the mixture was incubated at 30°C for 2 h in a dark shaker to obtain microbial cell/Fe_3_O_4_ biocomposites.

All biodegradation experiments were carried out in 100-ml flasks containing 10-ml MSM at 30°C on a reciprocal shaker at 180 rpm. In each experiment, 3,500 μg of carbazole was added to MSM, and the microbial cell/Fe_3_O_4_ biocomposites made by 2 ml mixture of Fe_3_O_4_ particle suspension and cell suspension served as biocatalysts. Additionally, the same amount of cells was conducted in the batch biodegradation experiment. All the subsequent experiments contained the same amount of carbazole and biocatalysts as above.

In the recycle experiments, after each batch of biodegradation, the microbial cell/Fe_3_O_4_ biocomposites were collected using a magnetic field, and then were washed thrice with MSM to remove the free cells. After the MSM was drained, 10 ml of fresh MSM containing carbazole was added to repeat the cycle. All experiments were performed in triplicate.

After each batch of biodegradation, the biodegradation mixture was added 20 ml ethanol, followed by centrifugation (12,000 rpm for 20 min) and filtration. Residual contents of carbazole were determined using High-performance liquid chromatography (HPLC). HPLC was performed with an Agilent 1100 series (Hewlett-Packard) instrument equipped with a reversed-phase C18 column (4.6 mm × 150 mm, Hewlett-Packard). The mobile phase was a mixture of methanol and deionized water (90:10, *v*/*v*) at a flow rate of 0.5 ml min^-1^, and carbazole was monitored at 254 nm with a variable-wavelength detector.

The size and morphology of magnetic nanoparticles and microbial cell/Fe_3_O_4_ biocomposite were determined by transmission electronic microscopy (TEM; JEM-100cx II, JEOL, Akishima-shi, Japan). The sample was prepared by evaporating a drop of properly diluted microbial cell/Fe_3_O_4_ biocomposite or nanoparticle suspension on a carbon copper grid. The morphology of free cells was determined using a scanning electron microscope (SEM; S-570, Hitachi, Chiyoda-ku, Japan). Magnetization curves for the magnetic immobilized cells were obtained with a vibrating sample magnetometer (MicroMag 2900/3900, Princeton Measurements Corp., Westerville, OH, USA).

## Results and discussion

### Characteristics of microbial cell/Fe_3_O_4_ biocomposites

**Figure 1:**
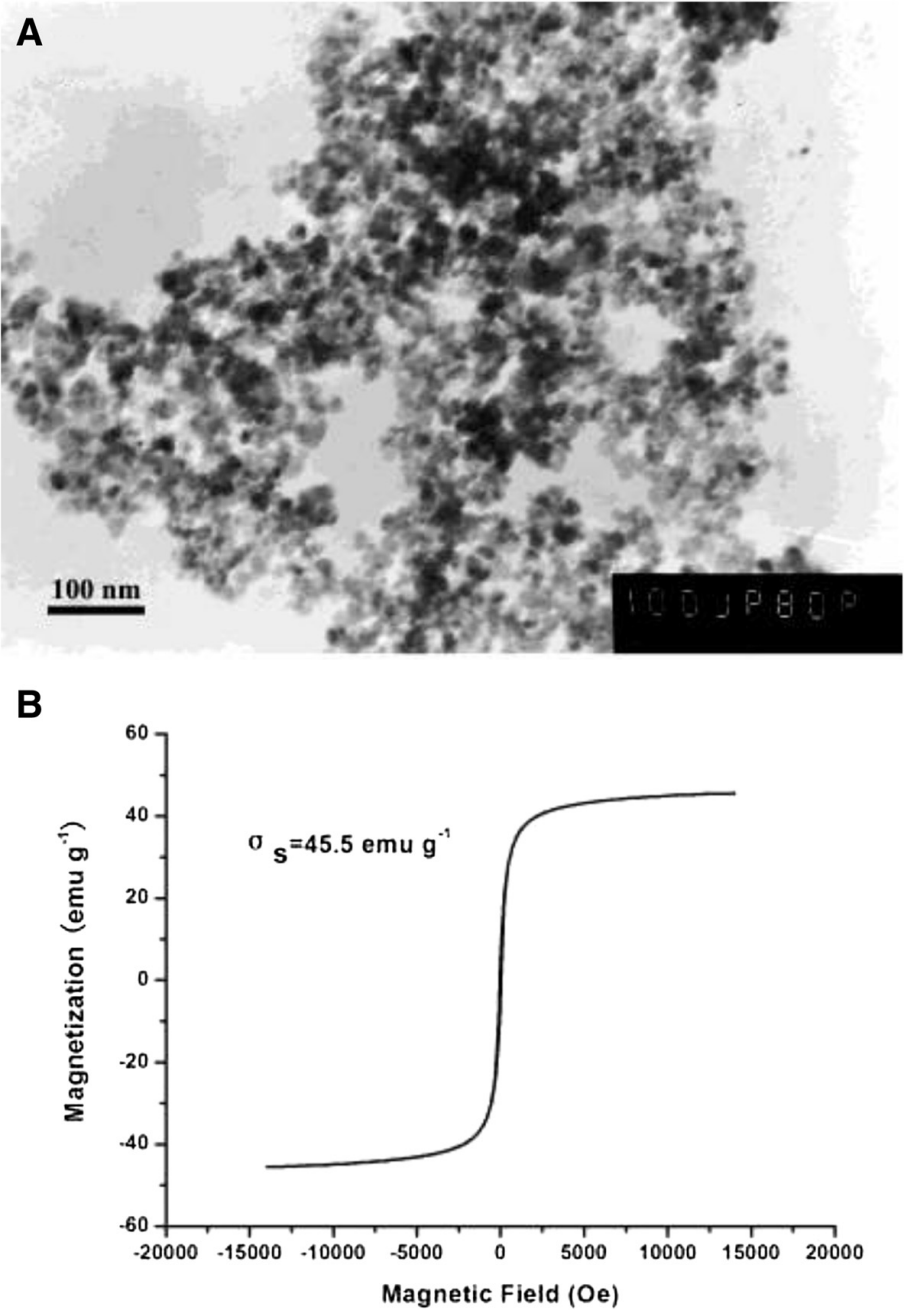
**The nature of Fe**_**3**_**O**_**4 **_**nanoparticles. A** is the TEM image of Fe_3_O_4_ (magnification × 100,000); **B** is the magnetic curve for Fe_3_O_4_ nanoparticles. (σ_s_, saturation magnetization; emu, electromagnetic unit; Oe, Oersted).

Among nanoparticles, nanoscale magnetite (Fe_3_O_4_) is of great interest as an immobilization carrier because of its biocompatibility, stability, large surface area, and super-paramagnetic properties [16,17]. In this study, an efficient microbial cell/Fe_3_O_4_ biocomposite was constructed by assembling Fe_3_O_4_ nanoparticles onto the surface of *Sphingomonas* sp. XLDN2-5 cells. Figure 1 showed the TEM images of Fe_3_O_4_ nanoparticles and their saturation magnetization. The average particle diameter of Fe_3_O_4_ nanoparticles was about 20 nm (Figure 1A), and their saturation magnetization was 45.5 emu · g^-1^ (Figure 1B), which provided the nanoparticles with super-paramagnetic properties.

**Figure 2:**
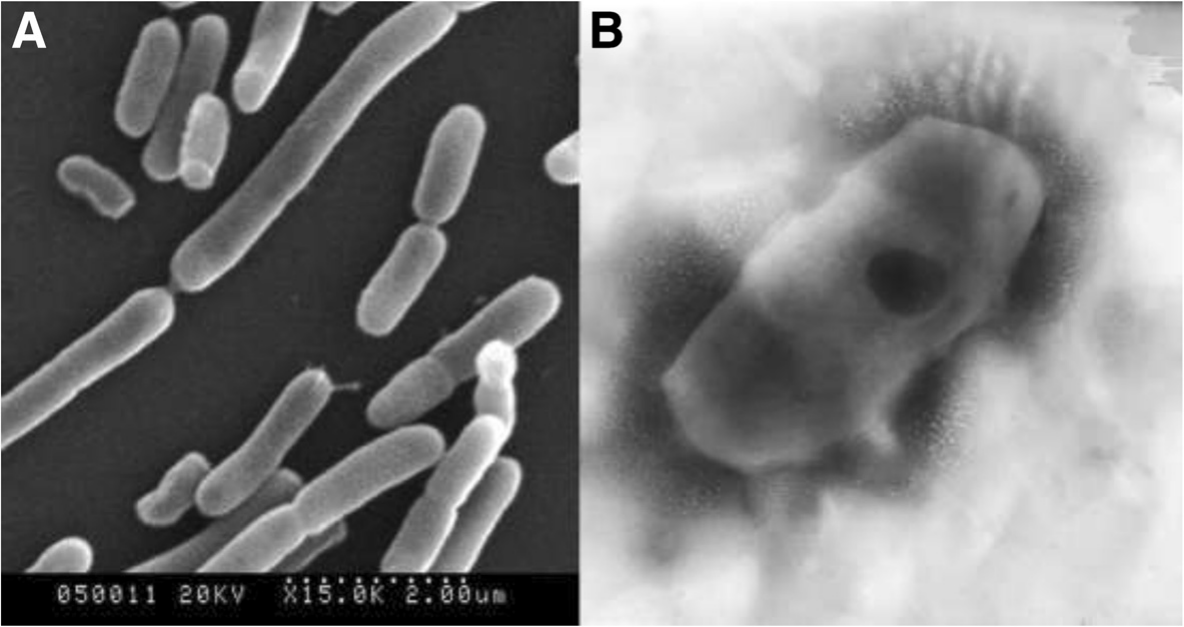
**The photograph of *Sphingomonas *sp. XLDN2-5. A** is the SEM image of *Sphingomonas* sp. XLDN2-5 (magnification × 15,000). **B** is the TEM image of microbial cell/Fe_3_O_4_ biocomposite (magnification × 36,000).

**Figure 3:**
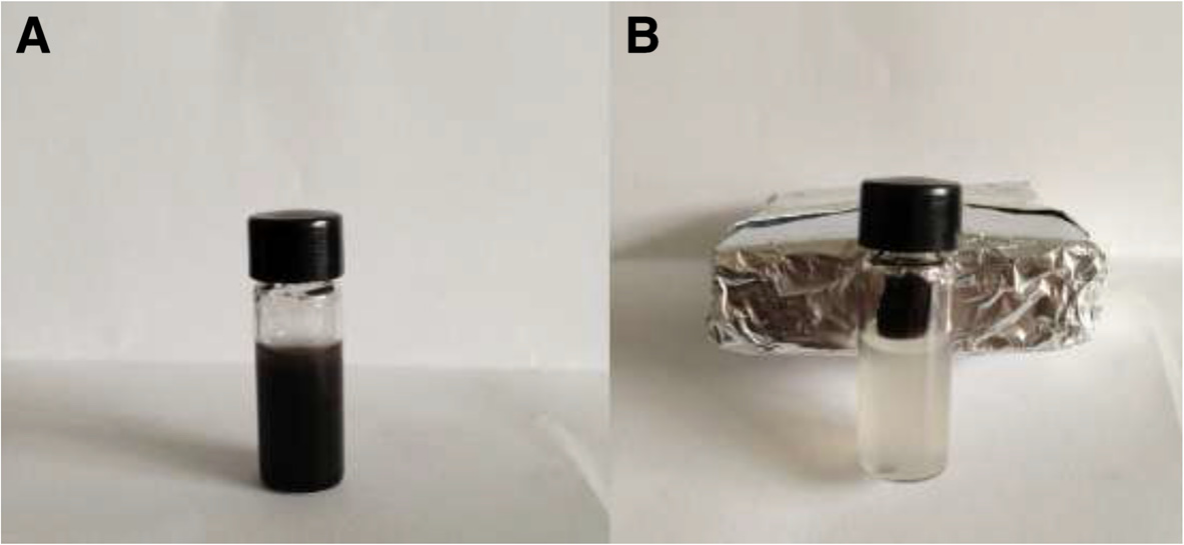
**Digital photo of microbial cell/Fe**_**3**_**O**_**4 **_**biocomposite suspension before (A) and after collection (B) using a magnetic field.**

Figure 2 shows the microbial cells of *Sphingomonas* sp. XLDN2-5 before and after Fe_3_O_4_ nanoparticle loading. The Fe_3_O_4_ nanoparticles were efficiently assembled on the surface of the microbial cell because of the large specific surface area and the high surface energy of the nanoparticles as shown in Figure 2B. It was clear that the size of the sorbent was much smaller than that of microbial cell, which was about a few micrometers as shown in Figure 2A. Due to the super-paramagnetic properties of Fe_3_O_4_ nanoparticle coating, the microbial cell/Fe_3_O_4_ biocomposite could be easily separated and recycled by external magnetic field as shown in Figure 3. When a magnet was touched to the side of a vial containing a suspension of microbial cell/Fe_3_O_4_ biocomposite (Figure 3A), the cells aggregated in the region where the magnet touched the vial (Figure 3B), which can be used with high efficiency in difficult-to-handle samples [14].

### Biodegradation activity and reusability of microbial cell/Fe_3_O_4_ biocomposites

**Figure 4:**
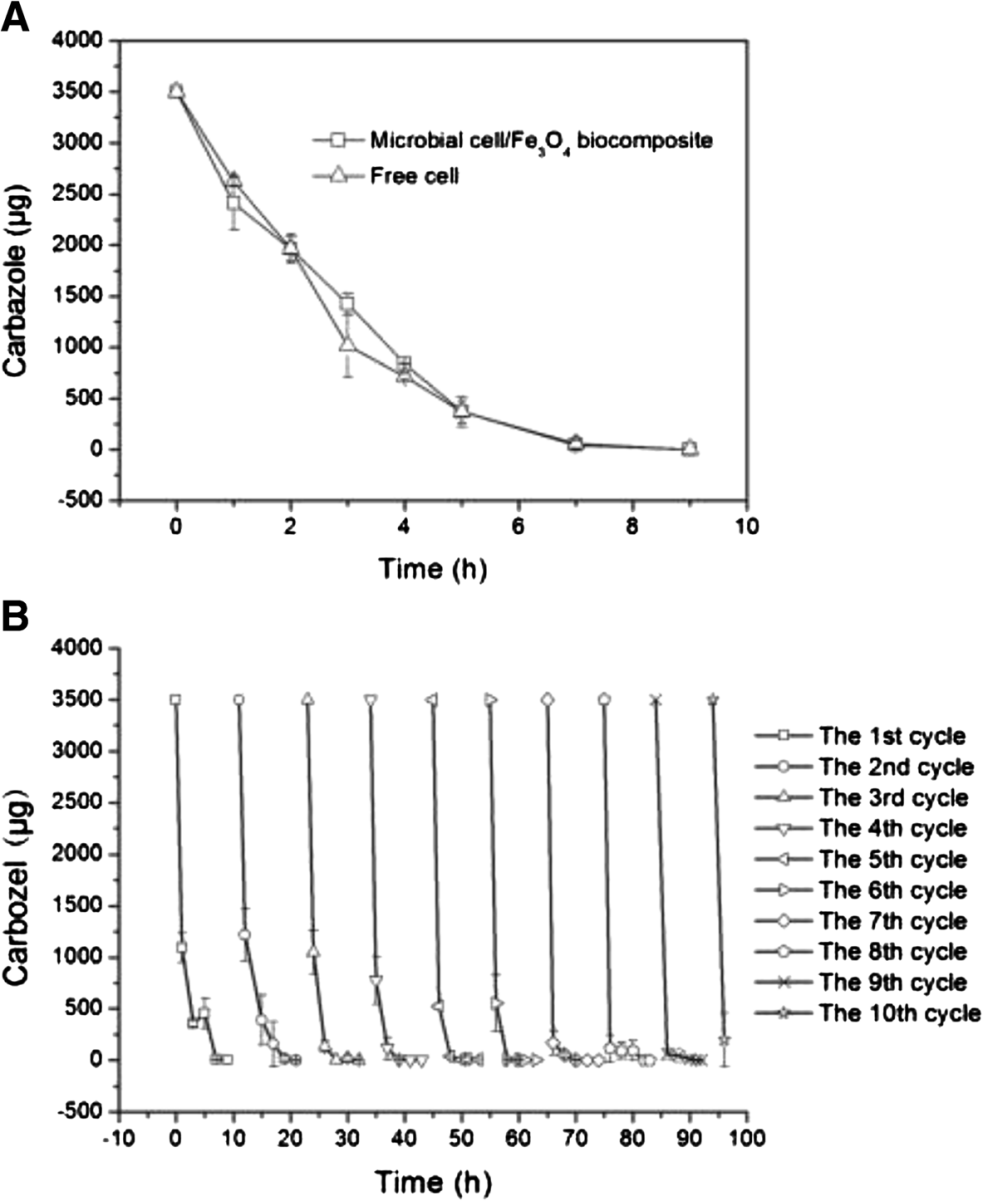
**The carbazole biodegradation by free cells and microbial cell/Fe**_**3**_**O**_**4 **_**biocomposites. A** is for carbazole biodegradation. **B** is for the reuse of microbial cell/Fe_3_O_4_ biocomposites.

With the purpose of understanding the biodegradation activity of the microbial cell/Fe_3_O_4_ biocomposite, the biodegradation rates of free cells and microbial cell/Fe_3_O_4_ biocomposite were tested at 30°C, respectively. Figure 4A showed that the microbial cell/Fe_3_O_4_ biocomposites had the same biodegradation activity as free *Sphingomonas* sp. XLDN2-5 cells. These results indicated that the Fe_3_O_4_ nanoparticle coating did not have a negative effect on the biodegradation activity of *Sphingomonas* sp. XLDN2-5. The reason may be that the coating layer of nanoparticles does not change the hydrophilicity of the cell surface due to biocompatibility of Fe_3_O_4_ nanoparticles [10,18], which are very important for the immobilization of microbial cells. Additionally, the effect of the coating layer on mass transfer is negligible because the structure of the coating layer is looser than that of the cell wall [11]. Thus, the microbial cell/Fe_3_O_4_ biocomposite could produce a system not limited by diffusional limitations [19].

In an industrial bioremediation process, the recycle of the biocatalysts could be an important factor that determines the effectiveness of degradation for a long time. The carbazole biodegradation activities of microbial cell/Fe_3_O_4_ biocomposite were tested repeatedly. Each test was performed until the carbazole was consumed completely. At the end of each test, the microbial cell/Fe_3_O_4_ biocomposites were collected by application of a magnetic field and then reused in another test. As shown in Figure 4B, from the first to the sixth cycle, 3,500 μg carbazole was completely consumed by microbial cell/Fe_3_O_4_ biocomposite in 9 h; from the seventh to the tenth cycle, the same amount of carbazole was completely consumed in only 2 h. It was clear that the biodegradation activity of microbial cell/Fe_3_O_4_ biocomposites increased gradually during the recycling processes, which may be due to that more microbial cells was immobilized by Fe_3_O_4_ nanoparticles with the microbial cell growth and reproduction. Additionally, carbazole can be quickly transferred to the biocatalyst surface where nanosorbents were located and resulted in the increase of biodegradation rate [10,14]. These results are different from other researchers' report which stated that the desulfurization activity of microbial cells coated by magnetite nanoparticles decreased gradually after a few test cycles [11].

## Conclusions

In conclusion, the microbial cell/Fe_3_O_4_ biocomposite was evaluated as a novel aspect of the industrialization of microbial cell immobilization. Moreover, magnetic (Fe_3_O_4_) nanoparticles have a large specific surface and super-paramagnetic properties, which not only reduced the mass transfer resistance of traditional immobilization method, but also facilitated the recovery of immobilized cells in the reuse process. Additionally, the recycle experiments demonstrated that the biodegradation activity of microbial cell/Fe_3_O_4_ biocomposites increased gradually during the recycling processes. These results indicated that magnetically modified microbial cells provide a promising technique for improving biocatalysts used in the biodegradation of hazardous organic compounds.

## Competing interests

The authors declare that they have no competing interests.

## Authors’ contributions

YL and XD designed the biodegradation experiments and carried out the characterization. CW and XL participated in Fe_3_O_4_ nanoparticles and microbial cell/Fe_3_O_4_ biocomposite fabrication. XW and PX made substantial contributions to the conception and design of this paper. XW and YL wrote the paper. All authors read and approved the final manuscript.
